# Participatory innovation for human papillomavirus screening to accelerate the elimination of cervical cancer

**DOI:** 10.1016/S2214-109X(20)30522-2

**Published:** 2021-05

**Authors:** Natalia M Rodriguez

**Affiliations:** Purdue University, West Lafayette, IN, USA

On Nov 17, 2020, WHO launched a global strategy to accelerate the elimination of cervical cancer.^1^ The strategy aims for all countries to achieve 90% human papillomavirus (HPV) vaccination coverage, 70% HPV screening coverage with a high-performance test, and 90% access to treatment for cervical pre-cancer and cancer by 2030. Despite being a preventable and treatable disease, cervical cancer remains one of the most serious threats to women’s lives globally, and is a marker of health disparity and limited health-care access.^2^ Screening is crucial to successful cervical cancer management because HPV infection is asymptomatic, progression of precancerous lesions is slow, and treatment of advanced disease can be challenging and costly. However, screening rates remain low in areas of poverty and marginalisation, even in high-income countries.^3^ In the USA, for example, the Healthy People 2020^4^ target of 93% cervical cancer screening coverage was not met, reaching only 80.5% in 2018. Screening rates decreased by 4% between 2008 and 2018, and screening coverage disparities persisted, including among uninsured people (61% screened), foreign-born immigrants (75% screened), people who completed less than high school education (71% screened), and people living in poverty (70% screened).^4^ The full impact of the COVID-19 pandemic on cancer screening is not yet known, but substantial drops in screening rates continue, with disproportionate effects on disadvantaged groups.^5^

Accompanying the global strategy, newly released WHO guidance^6^ on introducing and scaling up HPV testing for prevention and control of cervical cancer includes important considerations around building the right delivery model by “selecting testing strategies that will reach the target population” and “strongly considering community health care supported models using self-sampling”.^6^ To achieve cervical cancer screening coverage targets, simultaneous and strategic innovation is needed both technologically (new and better tools) and at delivery (better ways of implementing these tools).

WHO currently recommends HPV testing as a primary cervical cancer screening tool for women older than 30 years, where resources permit, and 2020 American Cancer Society guidelines^6,7^ included a shift to primary HPV testing, without cytology, as the preferred screening method. Only two devices for primary HPV screening are approved by the US Food and Drug Administration (FDA; cobas HPV [Roche Molecular Diagnostics, Pleasanton CA] and Onclarity HPV [BD Diagnostics, Sparks MD]), both of which require laboratory infrastructure and batch testing that can take hours. Rapid, single-use, molecular HPV tests could enable community-based testing with self-collected samples, circumventing many of the barriers to existing tests. Non-batch point-of-care tests, such as Xpert HPV (Cepheid, Sunnyvale CA), bring us a step closer to community-based testing. However, these tests still rely on laboratory and electricity-dependent platforms, and are not yet approved by the FDA.^8^ Machine-learning-based approaches and digital cytology, especially in the age of telehealth, also show promising preliminary results and should undergo urgent validation.^9^

However, technological innovation alone is rarely the answer. The process of successful uptake, adoption, and diffusion of new technologies into health systems, especially in low-resource settings, is poorly understood and often excludes the perspective of the end user or meaningful consideration of community, social structure, and cultural context. Human-centred design approaches to identify critical context-specific functional, systemic, and user requirements are essential. Furthermore, delivery strategies must consider the persistent sociocultural access barriers that exacerbate health disparities among low-income, uninsured, immigrant, and racial minority groups that are often marginalised and medically underserved. These include barriers related to the social determinants of health, low health literacy, reluctance to have a pelvic evaluation, stigma associated with sexually transmitted diseases like HPV, and cultural family dynamics potentiating risk of gender-based violence or abandonment after diagnosis.

Community-engaged approaches are needed to better understand and address these barriers. Community-based participatory research, for example, invites community stakeholder participation throughout the research process and has led to innovative delivery interventions that increased cervical cancer screening among high-risk populations, such as HPV self-sampling delivered by community health workers.^10^

As shown by decades of HIV and malaria testing in sub-Saharan Africa,^11,12^ community health workers can be instrumental to the acceptance and adoption of a screening test, but they must be appropriately trained and included, along with the target community, in the actual design of the technology. Moreover, implementation of an HPV test is more complex than are those for HIV or malaria, because a positive result does not necessarily indicate cervical cancer, but rather a higher risk that requires further testing. Successful adoption of a screening technology thus requires engagement of diverse stakeholders, including health-care providers, in both the design and implementation processes, to ensure follow-up testing and linkage to care.

Participatory approaches such as community-based participatory research and human-centred design are powerful tools that enable the technological and delivery innovations needed to implement the WHO global strategy, reach the most vulnerable worldwide, and eliminate cervical cancer in every context once and for all.
